# Salinity stress mitigation in tomato (*Solanum lycopersicum* L.): mechanisms, impacts and copper nanoparticle based solution

**DOI:** 10.3389/fpls.2026.1777876

**Published:** 2026-03-17

**Authors:** Rahul Anand, Shubhranshu Vardhan, Aruna Parihar, Deepesh Bhatt, Sandeep Arora

**Affiliations:** 1 Department of Molecular Biology and Genetic Engineering College of Basic Sciences & Humanities (CBSH), Govind Ballabh Pant University of Agriculture & Technology, Pantnagar, U.S. Nagar, Uttarakhand, India; 2 Department of Biotechnology, Shree Ramkrishna Institute of Computer Education and Applied Sciences, Sarvajanik University, Surat, Gujarat, India

**Keywords:** antioxidants, ionic homeostasis, copper sulfate nanoparticles, proteomics, salinity, Solanum lycopersicum

## Abstract

Salinity stress is a major abiotic factor that severely limits global crop productivity. It disturbs plant water relations, ion homeostasis and redox balance, leading to reduced plant growth and productivity. Conventional practices have only partially alleviated these constraints, especially in the rapidly expanding salt-affected areas, driven by climate change and unsustainable irrigation practices. In this context, copper-based nanoparticles (Cu-NPs) have emerged as promising nano-agrochemicals, capable of modulating multiple stress-responsive pathways. This review summarizes the current knowledge on the morpho-physiological, biochemical and molecular mechanisms implicated in salinity tolerance in tomato and critically evaluates how Cu based nanoparticles modulate cellular homeostasis to improve salt resilience. Evidence from physiological, biochemical and ionic studies indicates that Cu based nanoparticles stabilize cellular metabolism under saline conditions, by strengthening antioxidant defense, improve Na^+^ exclusion and K^+^ retention and protect photosynthetic performance. Proteomic investigations further reveal that in Cu-NP-treated tomato plants, the aforementioned cellular alternations are coordinated through stress signaling proteins and involve energy metabolism. Thus, providing a mechanistic basis for the observed phenotypic benefits. Genotype as well as concentration dependent responses emphasize that Cu-NP efficacy is maximized at intermediate doses under moderate stress, while excessive application can trigger copper toxicity and redox imbalance. The review also discusses potential environmental risks, regulatory gaps and standardization challenges associated with deployment of copper-based nanoparticles under field conditions. By integrating multi-scale evidence, the review provides a conceptual framework for rationally designing Cu-NP based interventions and identifies key research priorities for their safe and effective use in tomato cultivation.

## 1 Introduction

### 1.1 Global context of soil salinity

Soil salinity has emerged as one of the most pressing challenges confronting global agriculture in the 21st century. Approximately 20% of cultivated land and nearly 50% of irrigated agricultural areas worldwide are affected by salt accumulation, with these figures projected to increase due to climate change, inadequate irrigation practices and rising sea levels (Mishra et al., 2023). The economic implications are substantial, with annual crop losses attributed to salinity stress exceeding billions of dollars globally.

Tomato (*Solanum lycopersicum* L.), a glycophytic crop of immense economic and nutritional importance (**Table 1**), ranks among the most widely cultivated vegetables worldwide (Liu et al., 2022). Global tomato production has exhibited remarkable growth, reaching approximately 186 million metric tons in 2023 (FAOSTAT, 2024), with projections for 2024-25 indicating further expansion to approximately 196 million metric tons (FAOSTAT, 2025). China dominates global tomato production, contributing approximately 69-72 million metric tons (36-37% of global output), followed by India with 21-23 million metric tons, with United States America, Turkey, Egypt and Italy as other major producers (FAOSTAT, 2025). India’s tomato sector has witnessed substantial expansion over the past two decades, with the annual production increasing from approximately 10.5 million metric tons in 2001-02 to 21 million metric tons in 2021-22, representing a two-fold increase. Moreover, productivity has also improved from 16.29 t/ha in 2001-02 to 24.18 t/ha by 2021-22 (National Horticulture Board, 2023), with projections for 2024-25 indicating further enhancement to approximately 25-26 t/ha, reflecting its significance in national GDP (National Horticulture Board, 2025).

**Table 1:** Major bioactive compounds found in tomato and their health-related functions.

S.no.	Chemical compounds	Functions	Structure	Reference
1	Lycopene	Major carotenoid responsible for red color; strong antioxidant; reduces risk of cancer and cardiovascular diseases	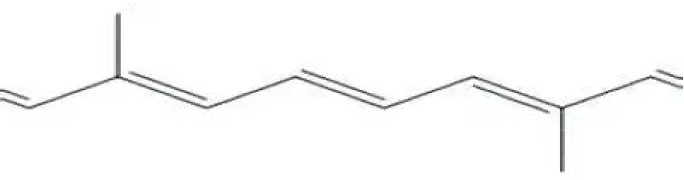	Rao and Rao (2007)
2	β-carotene	Provitamin A; supports vision, immunity and antioxidant activity	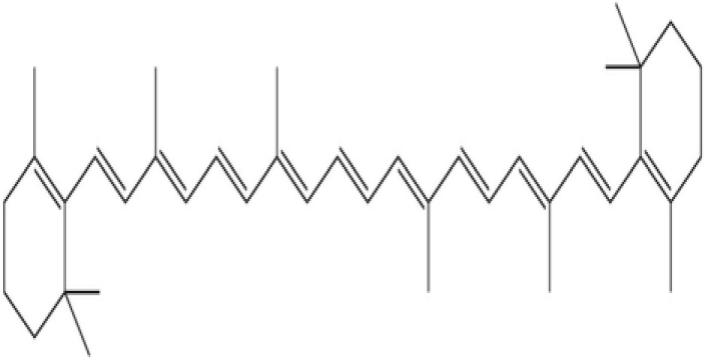	Fraser and Bramley (2004)
3	Ascorbic acid (Vitamin C)	Powerful antioxidant; enhances immunity and collagen synthesis	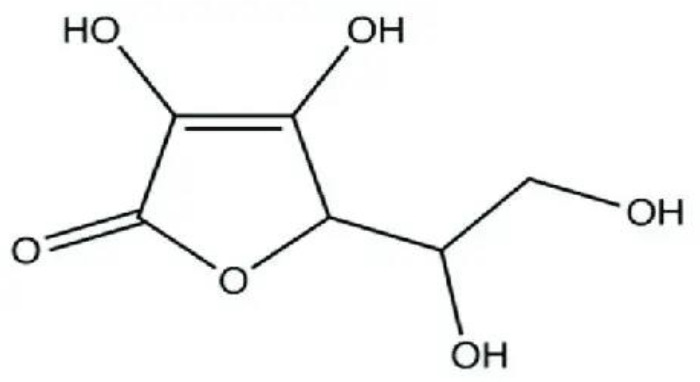	Davey et al. (2000)
4	Chlorogenic acid	Phenolic compound with antioxidant, anti-inflammatory and antimicrobial properties	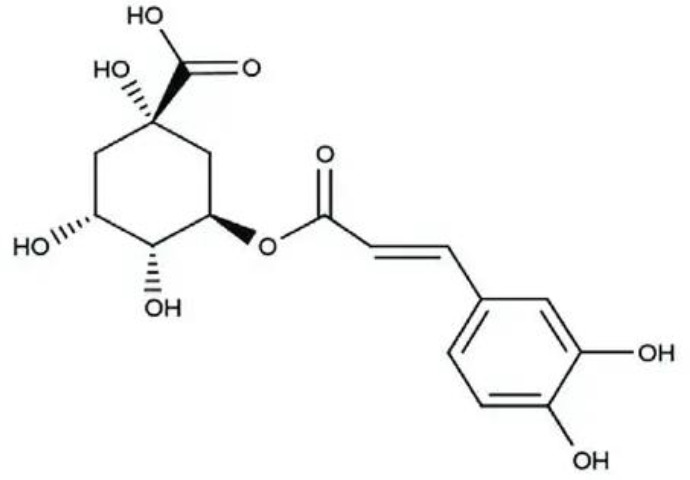	Niggeweg et al. (2004)
5	Quercetin	Flavonoid with antioxidant, anti-cancer and anti-inflammatory effects	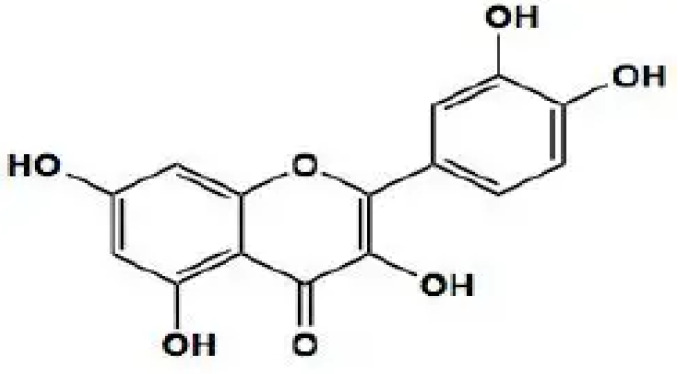	Slimestad and Verheul (2009)
6	Naringenin	Flavonoid involved in plant defense; antioxidant and anti-diabetic potential	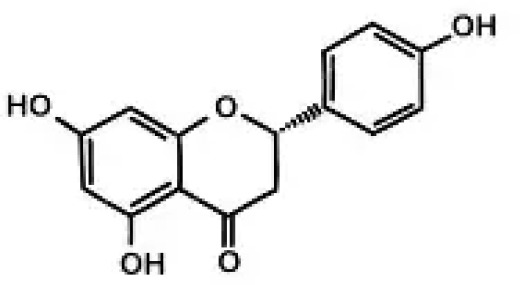	Stewart et al. (2000)
7	Citric acid	Major organic acid; contributes to flavor, pH regulation and metabolism	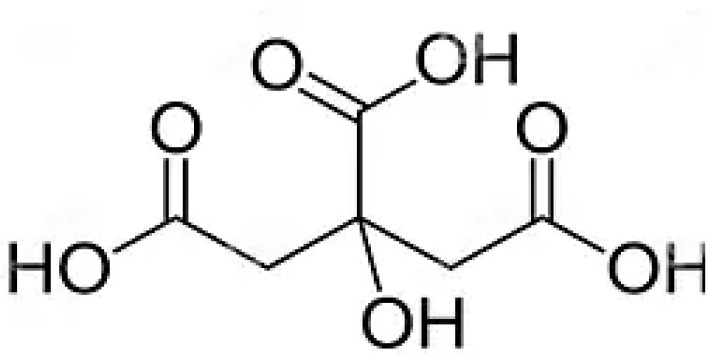	Pérez-Labrada et al. (2016)
8	Malic acid	Contributes to sour taste; involved in respiration and energy metabolism	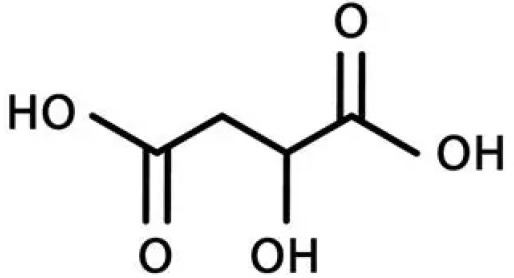	Chi et al. (2016)
9	Glutamic acid	Major amino acid responsible for umami flavor; important for protein synthesis	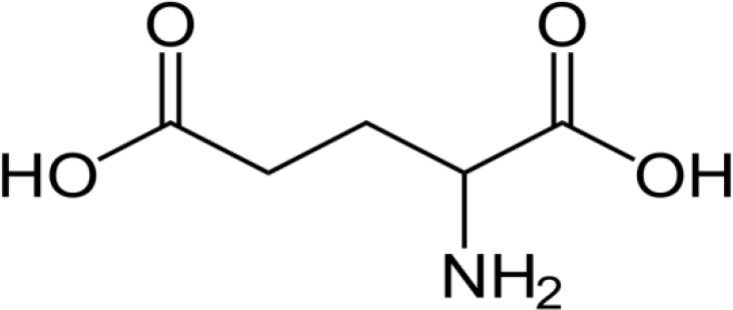	Lee et al. (2021)
10	Tomatine (α-tomatine)	Glycoalkaloid with antifungal and antibacterial defense role in plant	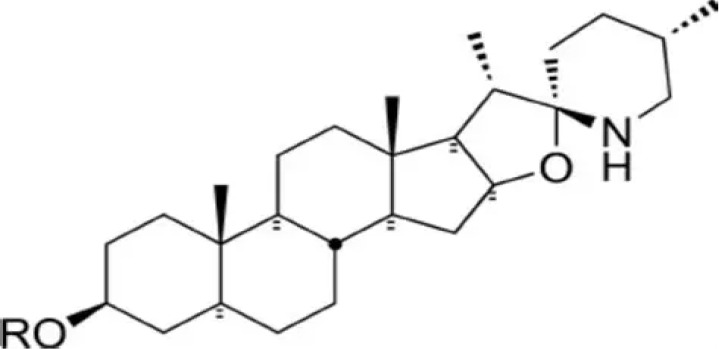	Friedman (2002)

Despite its global significance, tomato is classified as moderately sensitive to salt stress, with yield reductions (approximately 50%) becoming evident at soil electrical conductivity levels exceeding 2.5 dS/m (Cantore et al., 2012). This sensitivity threatens production in arid and semi-arid regions where irrigation water quality continues to decline, necessitating novel interventions to maintain productivity.

### 1.2 Role of nanotechnology in agriculture

The convergence of nanotechnology and agricultural science has opened unprecedented avenues for addressing crop stress challenges. Nanoparticles, defined as materials with at least one dimension between 1-100 nanometers, exhibit unique physicochemical properties that distinguish them from their bulk counterparts. These properties including high surface area-to-volume ratios, enhanced reactivity and improved cellular penetration-make nanoparticles particularly attractive for agricultural applications (Zaman et al., 2025).

Among various nanomaterials, copper-based nanoparticles have garnered significant attention due to copper’s essential role as a cofactor in numerous plant enzymes, including plastocyanin, cytochrome c oxidase and superoxide dismutase. The nanoscale form offers potential advantages over conventional copper fertilizers, including improved bioavailability, controlled release characteristics and targeted cellular delivery (Ahmed et al., 2026).

### 1.3 Rationale, scope and objective

Despite a growing body of literature reporting beneficial effects of nanoparticles under salinity stress, the underlying mechanisms remain fragmented across physiological, biochemical and molecular studies and a unified mechanistic framework specific to tomato is still lacking. In particular, recent proteomic and ionomic studies have generated large datasets describing nanoparticle-induced reprogramming of stress-responsive pathways, yet these findings are rarely integrated with classical physiological responses or varietal performance (Cherati and Khodakovskaya, 2025; Choudhary et al., 2025; Satti et al., 2022).

This review critically synthesizes current knowledge on salinity stress responses in tomato and evaluates the emerging role of copper-based nanoparticles as stress-mitigating agents. Rather than providing a purely descriptive overview, we emphasize mechanistic linkages across biological scales, integrating morphological, physiological, biochemical, proteomic and elemental evidence. Primary focus extends to proteomic insights that reveal coordinated regulation of antioxidant defense, ionic homeostasis, photosynthesis and primary metabolism under combined salinity and nanoparticle treatments. Key knowledge gaps are identified, including nanoparticle uptake and translocation pathways, long-term environmental fate and field-level performance under heterogeneous soil conditions. By addressing these gaps, this review aims to provide a conceptual foundation for the rational and responsible deployment of copper nanoparticle-based strategies in salt-affected tomato production systems.

## 2 Salinity stress in tomato: mechanisms and physiological impacts

Salinity stress in plants operates through two interconnected but temporally distinct mechanisms. The osmotic component manifests immediately upon salt exposure, reducing the water potential gradient between soil and roots, thereby impairing water uptake and causing physiological drought. This rapid response triggers stomatal closure, reduces leaf expansion and affects cellular turgor pressure (Atta et al., 2023).

The ionic component develops more gradually as sodium (Na^+^) and chloride (Cl^-^) ions accumulate in plant tissues. Excessive Na^+^ in the cytoplasm interferes with enzyme activities by displacing essential cations, particularly potassium (K^+^) and calcium (Ca²^+^), from their binding sites. This ionic interference disrupts protein structure, inhibits metabolic processes and compromises membrane integrity. Chloride accumulation exacerbates these effects through chlorophyll degradation and direct photosynthetic inhibition (Assaha et al., 2017).

The maintenance of K^+^/Na^+^ homeostasis emerges as a critical determinant of salt tolerance. Potassium is essential for enzyme activation, osmotic regulation and membrane polarization. Under saline conditions, Na^+^ competes with K^+^ for uptake through non-selective cation channels and transporters, leading to K^+^ deficiency even when soil K^+^ levels are adequate (Balasubramaniam et al., 2023).

### 2.1 Morphological and growth responses

#### 2.1.1 Vegetative growth inhibition

**Figure 1:**
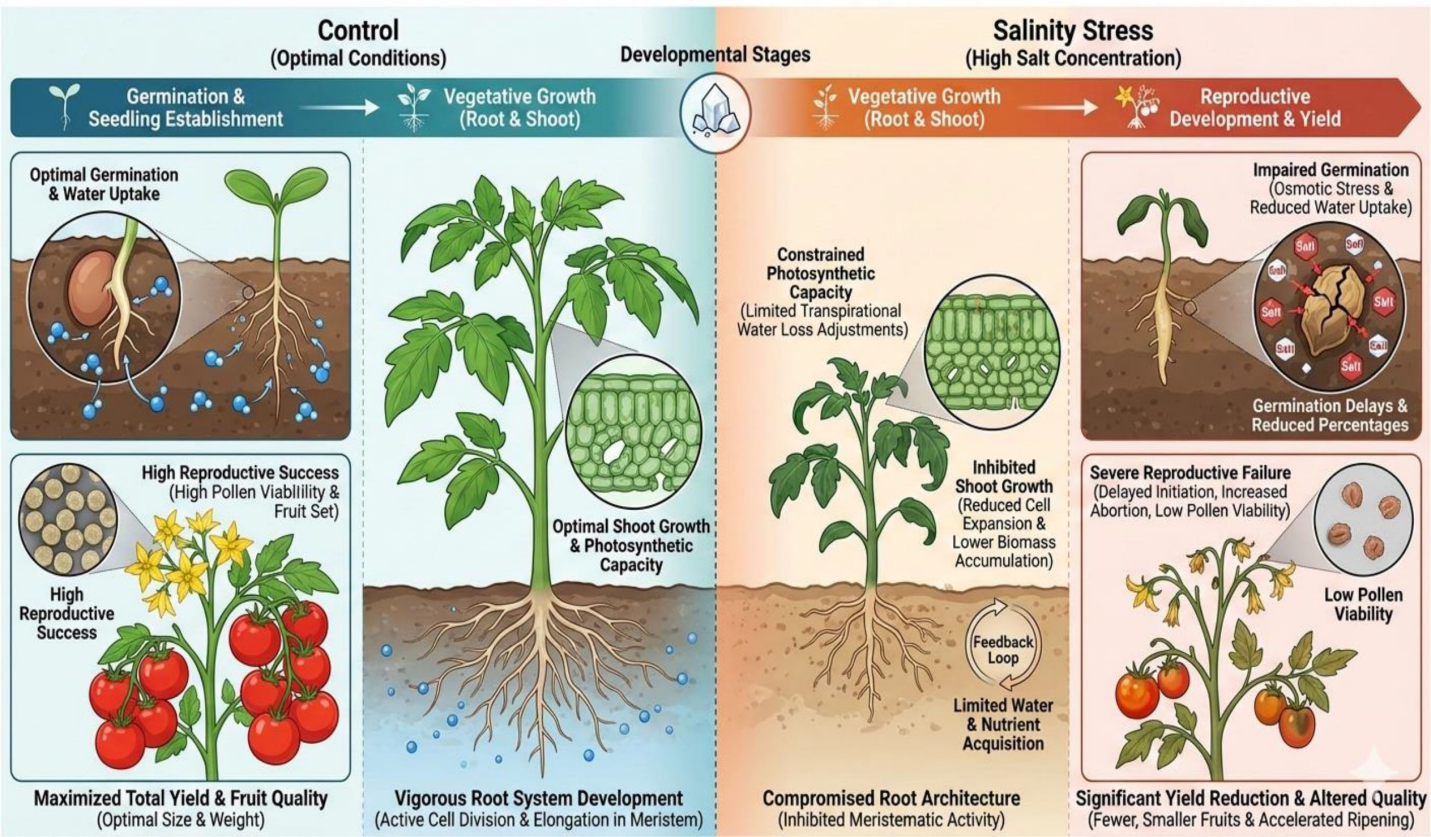
Impact of salinity stress on tomato.

Salinity stress profoundly impacts tomato growth from germination through reproductive development (**Figure 1**). Salt exposure impairs seed germination by reducing water uptake and imposing osmotic stress on the embryo, resulting in germination delays and reduced germination percentages (Guo et al., 2022).

Root system development suffers under saline conditions, with reductions in primary root length, lateral root formation and overall root biomass. These changes reflect reduced cell division and elongation in the root apical meristem, creating a feedback loop where compromised root architecture further limits water and nutrient acquisition (Sora et al., 2023).

Shoot growth shows parallel sensitivity, with reduced stem elongation, decreased leaf area and lower biomass accumulation. Leaves become smaller and thicker due to reduced cell expansion -adjustments that partially limit transpirational water loss but ultimately constrain photosynthetic capacity and growth potential (Al-Majdi and Al-Thuwaini, 2025).

#### 2.1.2 Reproductive development and yield

The reproductive phase exhibits heightened salt sensitivity. Flower initiation delays, abortion rates increase and pollen viability declines, collectively reducing fruit set. Fruits that do develop are often smaller, with reduced fresh weight and altered quality characteristics (Zhang et al., 2024). Total yield reductions result from fewer fruits per plant, reduced individual fruit size and accelerated ripening, with severity depending on stress intensity, duration and developmental timing (Zhang et al., 2022).

### 2.2 Physiological disruptions

#### 2.2.1 Photosynthetic impairment

Photosynthesis suffers from both stomatal and non-stomatal limitations under salt stress. Stomatal closure, an adaptive response to osmotic stress, reduces CO_2_ availability while conserving water. However, this response limits carbon fixation and growth (Guo et al., 2022).

Non-stomatal limitations become increasingly important under prolonged stress. Chlorophyll degradation reduces light harvesting capacity, while damage to photosystem II (PSII)-evident as reduced Fv/Fm ratios-impairs electron transport. Rubisco and Rubisco activase also suffer salt-induced inhibition, further constraining carbon fixation (Hameed et al., 2021).

#### 2.2.2 Water relations

Salinity disrupts plant water relations by reducing soil water potential and requiring osmotic adjustment for water extraction. Although plants accumulate compatible solutes to increase tissue osmotic potential, this adjustment often proves insufficient for maintaining adequate hydration. Relative water content (RWC) typically declines, driving stomatal closure and reducing leaf expansion rates (Polash et al., 2018).

#### 2.2.3 Interaction of salinity stress with calcium nutrition

Calcium plays a critical role in regulating plant stress responses and its interaction with other ions profoundly affects physiology and development of a plant. Under salt stress conditions, high concentrations of sodium (Na^+^) in the soil solution compete with calcium (Ca²^+^) for uptake at the root surface and for binding sites on cell membranes, effectively reducing calcium availability and transport in the plant (Balasubramaniam et al., 2023). This competition is inevitable, as both ions use similar transport pathways and the overwhelming presence of sodium disrupts the selective permeability of root cell membranes, impairing calcium absorption (Islam et al., 2023). Additionally, salt stress induces osmotic stress and ionic toxicity, which further compromises the plant’s vascular system and reduces transpiration rates, thereby limiting the mass flow of calcium to actively growing tissues (Ran et al., 2021). Since calcium is relatively immobile in the phloem and moves primarily through transpiration-driven xylem transport, organs with low transpiration rates-such as fruits, young leaves and root tips become particularly vulnerable to calcium deficiency under saline conditions (Islam et al., 2023). This calcium deficiency, exacerbated by salt stress, directly contributes to physiological disorders like blossom end rot (Topcu et al., 2022).

Blossom end rot (BER) is a physiological disorder commonly observed in tomatoes, peppers and squash, characterized by the development of dark, sunken, leathery lesions at the blossom end (distal end) of the fruit. The primary cause of BER is localized calcium deficiency in the developing fruit tissue, even when soil calcium levels may be adequate (Sethi et al., 2024). Calcium is essential for maintaining cell wall integrity and membrane stability; it forms calcium pectate cross-links in the middle lamella of cell walls, providing structural strength and preventing cell collapse (Topcu et al., 2022). When rapidly growing fruit tissues receive insufficient calcium, their cell walls become weak and unstable, leading to cellular breakdown and tissue collapse at the blossom end, where cells are dividing most rapidly and have the highest calcium demand. The disorder typically manifests during periods of rapid fruit development and is aggravated by environmental factors that reduce calcium transport to the fruit, including soil salinity, high temperatures, excessive nitrogen fertilization, water stress and inconsistent irrigation (Vinh et al., 2017). High soil salinity intensifies BER by reducing calcium uptake through ionic competition and by decreasing water availability, which reduces transpirational pull, the primary driving force for calcium movement to fruits (Kabir and Diaz-Perez, 2025; Maurya et al., 2024). Once the lesions develop, secondary infections by bacteria and fungi often occur, further degrading the affected tissue and rendering the fruit unmarketable (Kabiru and Yusuf, 2024; Osdaghi et al., 2021; Petrasch et al., 2019).

### 2.3 Oxidative stress and antioxidant responses

#### 2.3.1 Reactive oxygen species accumulation

Salinity stress triggers overproduction of reactive oxygen species (ROS), including superoxide radicals (O_2_•^-^), hydrogen peroxide (H_2_O_2_), hydroxyl radicals (•OH) and singlet oxygen (¹O_2_). While ROS serve signaling roles at low levels, salt stress disrupts electron transport chains in chloroplasts and mitochondria, causing excessive ROS generation (Abdelgawad et al., 2016).

These reactive molecules attack cellular components indiscriminately. Lipid peroxidation, particularly of membrane polyunsaturated fatty acids, compromises membrane integrity, with malondialdehyde (MDA) serving as a biomarker for oxidative damage. Protein oxidation causes amino acid modifications, peptide bond cleavage and enzyme inactivation. DNA oxidation results in mutations and impaired gene expression (Hasanuzzaman et al., 2021).

#### 2.3.2 Antioxidant defense systems

Plants employ coordinated enzymatic and non-enzymatic antioxidant systems to manage ROS and prevent oxidative damage.

##### 2.3.2.1 Enzymatic antioxidants

Superoxide dismutase (SOD) catalyzes the first defense line, converting superoxide radicals to H_2_O_2_ and O_2_. Multiple SOD isoforms exist in different cellular compartments (cytosol, chloroplasts, mitochondria), containing different metal cofactors (Cu/Zn-SOD, Mn-SOD, Fe-SOD). Salt stress typically increases SOD activity, though the magnitude varies with stress severity and genotype (Sarker and Oba, 2018).

Catalase (CAT), localized primarily in peroxisomes, decomposes H_2_O_2_ to water and oxygen. Its high substrate turnover rate makes it particularly effective when H_2_O_2_ concentrations are elevated (Alhudhaibi et al., 2024).

Ascorbate peroxidase (APX) reduces H_2_O_2_ using ascorbate as the electron donor, operating within the ascorbate-glutathione cycle. This cycle, involving APX, glutathione reductase (GR), dehydroascorbate reductase (DHAR) and monodehydroascorbate reductase (MDHAR), provides fine-tuned H_2_O_2_ control while regenerating antioxidant molecules. APX is crucial for chloroplast redox balance (Saleem et al., 2025).

Peroxidases (POX) catalyze H_2_O_2_-dependent oxidation of various substrates, participating in ROS scavenging, cell wall lignification, wound responses and pathogen defense (Abdelgawad et al., 2016).

##### 2.3.2.2 Non-enzymatic antioxidants

Ascorbate (vitamin C) directly scavenges multiple ROS species and serves as a substrate for APX. Glutathione functions as a redox buffer, maintains cellular redox status and participates in stress signaling. The ratio of reduced to oxidized forms reflects cellular redox state (Azeem et al., 2023).

Proline accumulation represents a multifunctional stress response, serving as an osmoprotectant, membrane stabilizer, free radical scavenger and possible molecular chaperone. Under salt stress, proline levels increase through enhanced biosynthesis and decreased degradation (Gao et al., 2023).

### 2.4 Varietal differences in salt tolerance

Tomato germplasm exhibits substantial genetic variation in salt tolerance, ranging from highly sensitive to moderately tolerant varieties. Understanding these differences illuminates tolerance strategies and guides breeding and intervention development (Rosca et al., 2023).

Salt-tolerant varieties like Hisar Arun demonstrate superior performance through multiple complementary mechanisms: enhanced Na^+^ exclusion from shoots, improved K^+^ retention maintaining favorable K^+^/Na^+^ ratios, effective vacuolar compartmentalization of toxic ions, stronger osmotic adjustment capacity through compatible solute accumulation and robust constitutive and inducible antioxidant systems (Hao et al., 2021).

In contrast, salt-sensitive varieties like PT3 lack effective Na^+^ exclusion, show poor K^+^ retention resulting in low K^+^/Na^+^ ratios, exhibit inadequate vacuolar compartmentalization allowing cytoplasmic Na^+^ accumulation, demonstrate limited osmotic adjustment capacity and possess weaker antioxidant defenses leading to greater oxidative damage and membrane injury (Okut and Palta, 2025).

## 3 Copper nanoparticles: properties and characterization

### 3.1 Physicochemical properties

Copper nanoparticles exhibit distinctive properties that underlie their biological activities and agricultural applications. Their nanoscale dimensions (1-100 nm) confer high surface area-to-volume ratios, dramatically increasing surface atom proportions and enhancing reactivity and bioavailability compared to bulk copper. Quantum confinement effects alter electronic properties, affecting optical, catalytic and redox characteristics. Surface chemistry-including functional groups, coatings and oxidation states-significantly influences stability, bioavailability and biological interactions (Zaman et al., 2025).

Copper nanoparticles exist in various oxidation states: metallic copper (Cu^0^), cuprous oxide (Cu_2_O) and cupric oxide (CuO). Oxidation state affects reactivity, with metallic and cuprous forms generally showing higher biological activity. However, metallic copper nanoparticles require stabilization to prevent oxidation (Čarapar et al., 2025).

### 3.2 Characterization

**Figure 2:**
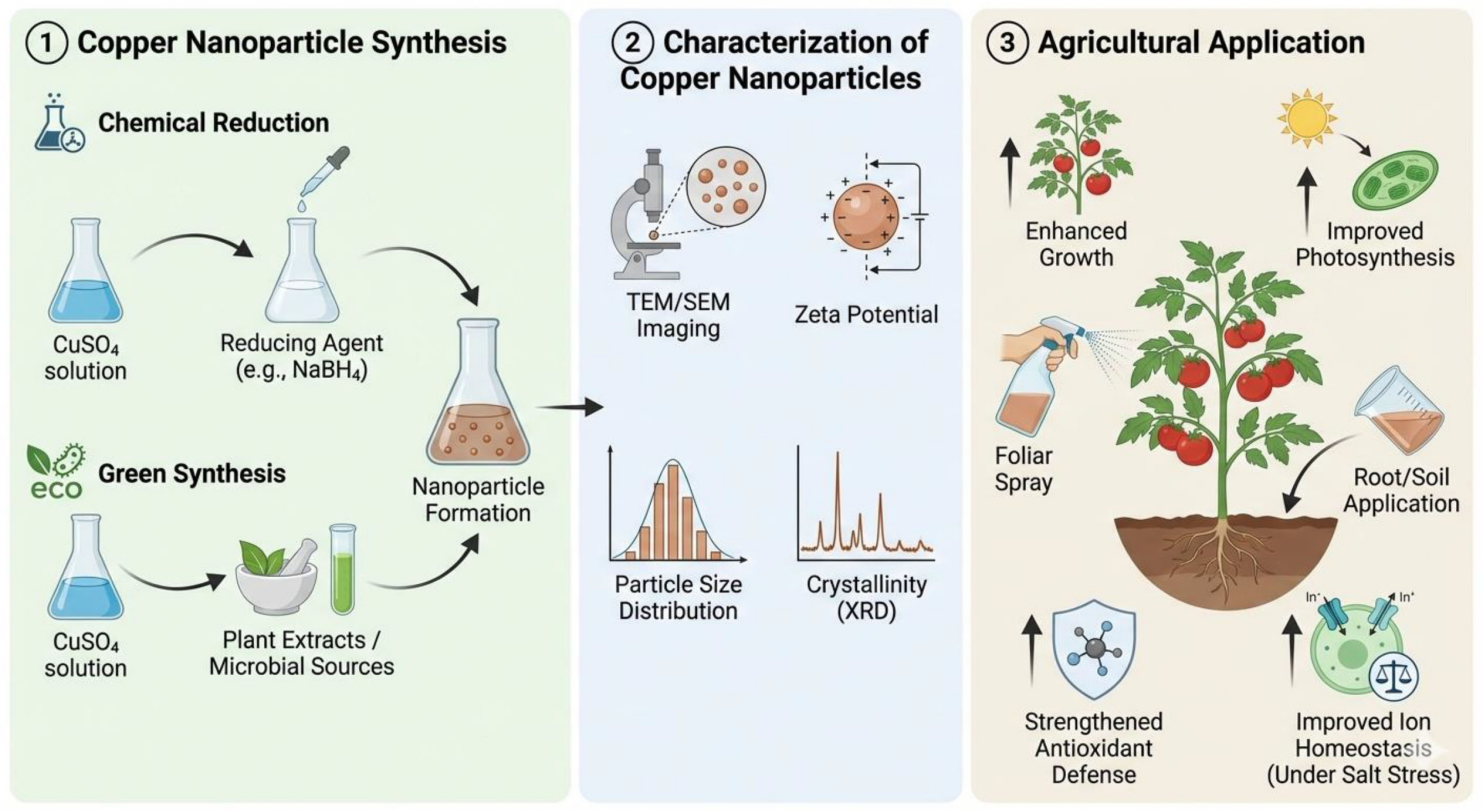
Workflow of copper nanoparticle synthesis, physicochemical characterization and agricultural applications demonstrating enhanced plant growth, photosynthesis and stress alleviation.

Proper characterization is essential for understanding nanoparticle properties and predicting biological activities (**Figure 2**). Key techniques include transmission electron microscopy (TEM) and scanning electron microscopy (SEM) for direct visualization of size, shape and distribution; dynamic light scattering (DLS) for hydrodynamic diameter measurement; X-ray diffraction (XRD) for crystal structure and oxidation state identification; Fourier-transform infrared spectroscopy (FTIR) for surface functional group identification; zeta potential measurements for surface charge assessment and stability prediction; and energy-dispersive X-ray spectroscopy (EDS) and inductively coupled plasma optical emission spectrometry (ICP-OES) for elemental composition quantification (Mourdikoudis et al., 2018; Santhoshkumar et al., 2017).

## 4 Copper nanoparticle-mediated stress alleviation: mechanisms and effects

### 4.1 Enhancement of antioxidant defense systems

Copper nanoparticles exert profound effects on antioxidant systems, representing a primary stress protection mechanism.

SOD activity can increase by 30-50% compared to salt-stressed controls, improving superoxide radical conversion. This enhancement involves multiple factors: nanoparticles provide bioavailable copper for SOD cofactors, may induce SOD gene expression through redox signaling and protect existing SOD proteins from oxidative damage (Pérez-Labrada et al., 2019).

CAT activity shows similar enhancement, accelerating H_2_O_2_ decomposition. APX activity increases while maintaining ascorbate pools, strengthening the ascorbate-glutathione cycle. POX activities also increase, contributing to H_2_O_2_ detoxification while supporting lignin formation and cell wall strengthening. The coordinated upregulation indicates comprehensive antioxidant network activation rather than isolated enzyme effects (Hernandez-Hernandez et al., 2019).

Beyond enzymatic systems, proline accumulation increases significantly under nanoparticle treatment subjected to salinity stress, providing improved osmoprotection and free radical scavenging. The ascorbate-glutathione system shows favorable modulation with maintained or enhanced levels of reduced forms, indicating better cellular redox balance (Gohari et al., 2021). Beyond enzymatic systems, copper nanoparticles influence non-enzymatic antioxidant pools. Proline accumulation increases significantly under combine salt stress and nanoparticle treatment, exceeding levels observed with salt stress alone. This enhanced proline accumulation results from upregulated biosynthesis and possibly reduced degradation, providing improved osmoprotection and free radical scavenging capacity (Shadidijazi and Taspinar, 2025). The ascorbate-glutathione system shows favorable modulation under nanoparticle treatments, with maintained or enhanced levels of reduced ascorbate and glutathione. The ratio of reduced to oxidized forms of these antioxidants improves, indicating better cellular redox balance. This improvement reflects both direct effects of nanoparticles on antioxidant biosynthesis and indirect effects through reduced oxidative stress (Li et al., 2022).

### 4.2 Regulation of ionic homeostasis

A critical mechanism through which copper nanoparticles enhance salt tolerance involves modulation of ion transport systems.

#### 4.2.1 Sodium management

Nanoparticles influence membrane transporters involved in Na^+^ homeostasis. The Salt Overly Sensitive (SOS) pathway shows enhanced activity: upon salt stress, calcium signals activate the SOS3-SOS2 complex, which phosphorylates and activates SOS1 (a plasma membrane Na^+^/H^+^ antiporter), promoting Na^+^ efflux. Copper nanoparticles appear to enhance this pathway, reducing cytoplasmic Na^+^ accumulation (Singh et al., 2025; Younis and Mansour, 2023).

**Figure 3:**
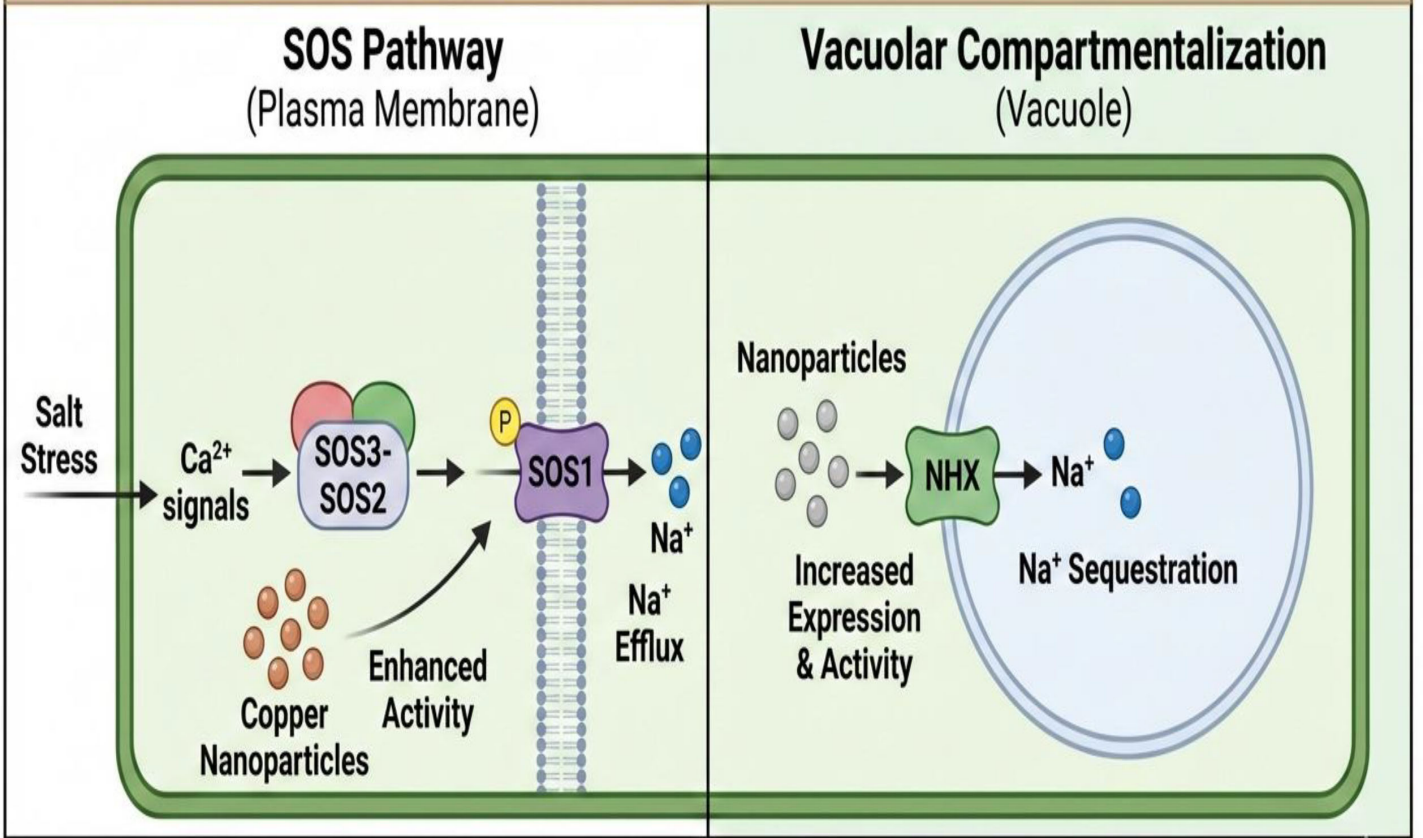
Copper nanoparticle mediated sodium homeostasis mechanism in plants under salt stress.

Vacuolar compartmentalization represents another enhanced strategy (**Figure 3**). The vacuolar Na^+^/H^+^ exchanger (NHX) transports Na^+^ from cytoplasm into vacuoles, sequestering toxic ions. Nanoparticle treatments increase NHX expression and activity, improving ionic compartmentalization and maintaining lower cytoplasmic Na^+^ concentrations (Jabeen et al., 2022).

#### 4.2.2 Potassium retention

Maintaining adequate K^+^ uptake under saline conditions is crucial, as K^+^ is essential for enzyme activation, osmotic regulation and electrical neutrality. Copper nanoparticles enhance K^+^ uptake mechanisms, improving the K^+^/Na^+^ ratio-a central indicator of salt tolerance. The improvement results from both reduced Na^+^ accumulation and improved K^+^ uptake through enhanced transporter activity (Khan et al., 2025).

ICP-OES elemental analysis confirms these changes; root and shoot Na^+^ concentrations are consistently lower in nanoparticle-treated plants, while K^+^ concentrations are maintained or elevated. Calcium homeostasis is also improved, supporting membrane integrity and stress signal transduction (Rahul et al., 2025).

### 4.3 Photosynthetic protection and metabolic support

#### 4.3.1 Chlorophyll protection

Chlorophyll degradation under salt stress represents a major photosynthetic limitation. Copper nanoparticles protect chlorophyll molecules from oxidative damage through both direct antioxidant effects and indirect protection via enhanced enzymatic systems. Chlorophyll fluorescence measurements reveal that Fv/Fm ratios (representing maximum PSII quantum efficiency) remain closer to optimal levels (0.8) under nanoparticle treatments, indicating better photosynthetic machinery preservation (Loudari et al., 2020).

#### 4.3.2 Gas exchange improvement

Copper nanoparticles modulate stomatal behavior, maintaining adequate CO_2_ uptake while controlling water loss. Net photosynthetic rate shows significant improvement through both stomatal effects (increased conductance) and non-stomatal effects (enhanced enzymatic capacity, protection of Rubisco and Calvin cycle enzymes). Rather than suppressing transpiration excessively, nanoparticles optimize water use efficiency-the ratio of carbon assimilation to water loss (Shaikhaldein et al., 2024).

#### 4.3.3 Metabolic maintenance

Salt stress disrupts primary metabolic pathways. Copper nanoparticles help maintain metabolic flux through glycolysis, TCA cycle and associated pathways (Shafiq et al., 2024; Li et al., 2020). Proteomic analyses reveal better preservation or enhanced expression of metabolic enzymes. Reduced oxidative stress prevents enzyme inactivation, while improved ionic homeostasis maintains conditions conducive to enzyme activity (Kosová et al., 2018). Enhanced energy production through protected mitochondrial function ensures adequate ATP supply. Nanoparticles also influence secondary metabolism, with phenylalanine ammonia-lyase (PAL) showing increased activity, supporting enhanced phenolic antioxidant production (Ning et al., 2025).

## 5 Proteomic insights into nanoparticle-mediated stress tolerance

Proteomic profiling using techniques such as two-dimensional gel electrophoresis, liquid chromatography-mass spectrometry (LC-MS) and isobaric tags for relative and absolute quantitation (iTRAQ) has revealed protein-level changes underlying nanoparticle-mediated stress tolerance, identifying hundreds of differentially expressed proteins (DEPs) in tomato plants subjected to salt stress with and without nanoparticle treatments (Chen et al., 2009).

### 5.1 Major protein categories affected

#### 5.1.1 Stress response and defense proteins

Heat shock proteins (HSPs) function as molecular chaperones, assisting protein folding, preventing aggregation and facilitating refolding or degradation of damaged proteins. Multiple HSP families (HSP70, HSP90, small HSPs) show upregulation in nanoparticle-treated plants, indicating enhanced protein quality control (Athar et al., 2022). Late embryogenesis abundant (LEA) proteins and dehydrins also increase in abundance, stabilizing membranes and enzymes under water-deficit conditions (Pakar et al., 2025).

#### 5.1.2 Antioxidant and redox-related proteins

Proteomic confirmation of SOD, catalase and peroxidase upregulation provides molecular support for biochemical assay results. Proteins involved in the ascorbate-glutathione cycle and glutathione metabolism show coordinated upregulation. Thioredoxin and peroxiredoxin systems, providing thiol-based redox regulation and H_2_O_2_ sensing, also show altered expression (Manaa et al., 2013).

#### 5.1.3 Photosynthesis-related proteins

Photosystem II subunits, particularly the D1 protein forming the PSII reaction center, show better maintenance under nanoparticle treatments. Rubisco and Rubisco activase show responsive expression and post-translational modifications. Chlorophyll-binding proteins and light-harvesting complex components suggest better chloroplast function, reflecting integrated photosynthetic protection (Chen et al., 2022).

#### 5.1.4 Ion transport proteins

Plasma membrane and tonoplast H^+^-ATPases show increased abundance, energizing secondary active transport. Na^+^/H^+^ antiporters (SOS1, NHX family) and K^+^ transporters show altered expression consistent with improved ionic homeostasis (Guo et al., 2022).

#### 5.1.5 Metabolic enzymes

Glycolytic and TCA cycle enzymes show maintained or increased abundance, supporting energy production. Amino acid metabolism proteins, particularly those in proline biosynthesis, show upregulation consistent with enhanced compatible solute accumulation (Cherati and Khodakovskaya, 2025; Chen et al., 2009).

### 5.2 Gene ontology and KEGG pathway analysis

Biological Process enrichments include oxidative stress response, osmotic stress response, ion homeostasis, cellular homeostasis, photosynthesis, carbohydrate metabolism, nitrogen metabolism, protein folding, proteolysis and signal transduction (particularly ABA and calcium signaling) (Keshishian et al., 2018).

Molecular Function enrichments include antioxidant activity, oxidoreductase activity, protein binding, ion binding, catalytic activities (carbohydrate and amino acid metabolism), transporter/channel activity, protein kinase activity and transcription factor activity (Wang et al., 2022).

Cellular Component enrichments highlight chloroplast, mitochondrion, cell wall, membrane (plasma membrane and tonoplast) and ribosome components (Keshishian et al., 2018).

Photosynthesis pathways (light reactions and Calvin cycle) shows coordinated regulation of photosystems I and II, cytochrome b6f complex, ATP synthase and Calvin cycle enzymes (Degen and Johnson, 2024). Antioxidant pathways (ascorbate-glutathione cycle, glutathione metabolism, peroxidase pathways) and phenylpropanoid biosynthesis show enrichment (Ikram et al., 2025; Foyer and Kunert, 2024). Stress signaling pathways, including MAPK cascades (crucial for translating stress perception into cellular responses, linked to ABA signaling) and calcium signaling, show enrichment (Ikram et al., 2025; Zhang et al., 2023). Metabolic pathways including glycolysis/gluconeogenesis, TCA cycle, amino acid metabolism and nitrogen metabolism are also affected (Tao et al., 2022; Ren et al., 2024; Chinnusamy et al., 2005).

### 5.3 Protein-protein interaction networks

Protein-Protein Interaction (PPI) analysis reveals network relationships between stress-responsive proteins, identifying hub proteins with high connectivity that represent key regulatory nodes. Common hubs include transcription factors regulating stress-responsive gene expression, protein kinases modulating numerous targets, metabolic enzymes integrating multiple pathways and heat shock proteins maintaining protein homeostasis. The network structure reveals functional modules (antioxidant defense, photosynthesis, ion transport) and connections between modules indicating crosstalk between stress response systems (Athar et al., 2022; Melicher et al., 2022; Wang et al., 2023; Anwar and Zhang, 2022; Hosseini et al., 2023).

## 6 Elemental composition and concentration-dependent effects

### 6.1 Copper accumulation and distribution

ICP-OES analysis reveals that copper concentrations increase in roots and shoots following nanoparticle treatment, though accumulation patterns vary with application method, concentration and tissue. Root tissues typically accumulate higher copper than shoots, indicating limited translocation. This distribution suggests transformation to less mobile forms or root sequestration. Despite limited translocation, bioavailable copper contributes to shoot enzyme cofactor pools, as evidenced by increased SOD activity (Perez-Labrada et al., 2019; Peshkova et al., 2025).

Copper tissue concentrations increase with applied nanoparticle concentration, though not always linearly. At low concentrations, accumulation is modest and within physiological ranges. At higher concentrations, accumulation is more substantial, requiring monitoring to ensure benefits outweigh toxicity risks (Peshkova et al., 2025).

### 6.2 Ionic balance and micronutrient status

Nanoparticle treatments consistently reduce Na^+^ and Cl^-^ accumulation while maintaining or elevating K^+^, Ca²^+^ and Mg²^+^ concentrations. The improved K^+^/Na^+^ ratio serves as a key salt tolerance indicator. Iron, manganese, zinc and other micronutrients may also show improved uptake or distribution, contributing to enhanced plant health (Alam et al., 2022; Pérez-Labrada et al., 2019).

Beyond copper, other micronutrients show altered accumulation patterns under nanoparticle treatments. Iron, manganese and zinc-essential micronutrients for numerous enzymatic processes-may show improved uptake or distribution. Silicon nanoparticles, when used in combination or comparison studies, demonstrate improvements in multiple micronutrient levels, though mechanisms remain incompletely understood (Yan et al., 2024; Alam et al., 2022).

The overall improvement in nutrient balance under nanoparticle treatments contributes to enhanced plant health and stress tolerance. Adequate micronutrient supply ensures that enzymatic systems function optimally, supporting both growth and stress defense processes (Alam et al., 2022).

## 7 Concentration-dependent effects and optimization

### 7.1 Salt stress gradients

The effectiveness of copper nanoparticles varies with salt stress intensity, revealing important insights for practical applications and mechanistic understanding.

#### 7.1.1 Low salinity stress

At low salt concentrations, stress effects are relatively mild, with plants showing manageable reductions in growth and physiological parameters. Under these conditions, nanoparticle treatments provide additive benefits, primarily through enhanced antioxidant systems and maintained photosynthetic efficiency. The stress response mechanisms are not overwhelmed, allowing nanoparticles to optimize existing defense systems (Rosca et al., 2023).

#### 7.1.2 Moderate salinity stress

Intermediate salt concentrations represent the conditions where copper nanoparticles show maximum effectiveness. At this stress level, multiple stress response pathways are activated and the plant’s endogenous defense mechanisms are approaching their limits. Nanoparticle interventions provide critical support, enhancing antioxidant systems, improving ion homeostasis and protecting photosynthetic machinery.

The synergy between activated stress responses and nanoparticle effects produces substantial improvements in stress tolerance. Growth parameters, photosynthetic rates and yield components show significant improvement compared to salt-stressed controls, approaching or sometimes matching non-stressed control levels. This effectiveness window makes medium salinity the most relevant condition for demonstrating nanoparticle potential (Zhou et al., 2025).

#### 7.1.3 High salinity stress

Severe salt stress overwhelms plant defense mechanisms, causing dramatic reductions in growth, photosynthesis and survival. Under these extreme conditions, nanoparticle effectiveness is limited, though some protective effects remain evident. The limitation reflects the severity of osmotic and ionic stress exceeding the capacity of any single intervention to fully compensate (Guo et al., 2022).

Even with limited effectiveness, nanoparticles provide measurable benefits at high salinity, including reduced oxidative damage, partial maintenance of photosynthetic capacity and improved survival rates. These effects, while insufficient to restore normal growth, may be valuable in highly saline environments where crop survival itself is uncertain (Rosca et al., 2023).

### 7.2 Nanoparticle concentration optimization

#### 7.2.1 Low concentrations of CuSO_4_ nanoparticle

Low nanoparticle concentrations provide subtle but significant improvements in stress tolerance without causing toxicity symptoms. These concentrations are suitable for preventive applications and long-term stress management. Benefits include mild antioxidant system enhancement, slight improvements in photosynthetic efficiency and better growth compared to untreated stressed plants (Perez-Labrada et al., 2019; Hernandez-Hernandez et al., 2019).

The safety margin at low concentrations is high, with minimal risk of copper toxicity or other adverse effects. This makes low-concentration applications attractive for sensitive varieties or situations where conservative approaches are warranted. However, the magnitude of benefit may be insufficient under severe stress conditions (Li et al., 2024).

#### 7.2.2 Medium concentration

Intermediate nanoparticle concentrations often provide optimal benefits, balancing effectiveness with safety. At these concentrations, robust stress response activation occurs, including substantial antioxidant system enhancement, significant ion homeostasis improvement and marked photosynthetic protection (Perez-Labrada et al., 2019).

Growth and yield responses at medium concentrations show substantial improvements over stressed controls, with parameters often approaching non-stressed levels. The copper accumulation at these concentrations remains within acceptable ranges, with no evidence of toxicity. This effectiveness-safety balance makes medium concentrations attractive for practical applications (Rahul et al., 2025).

#### 7.2.3 High concentrations

High nanoparticle concentrations may provide maximum stress protection but carry increased risk of toxicity. At higher concentrations, copper accumulation in tissues becomes substantial, requiring careful monitoring. While some studies report benefits at these concentrations, others note diminishing returns or even negative effects due to copper toxicity (Abbasirad. and Ghotbi-Ravandi, 2025).

The toxicity risk at high concentrations manifests as leaf chlorosis, reduced growth and oxidative stress-ironically, symptoms resembling those nanoparticles are meant to alleviate. This toxicity reflects excessive copper accumulation interfering with enzyme functions and generating pro-oxidant effects. The narrow window between beneficial and toxic effects at high concentrations limits practical utility (Petrus et al., 2025).

## 8 Varietal responses and interaction effects

### 8.1 Salt-tolerant varieties

Salt-tolerant varieties like Hisar Arun possess inherent stress tolerance mechanisms, including effective Na^+^ exclusion, strong K^+^ retention, robust antioxidant systems and efficient osmotic adjustment (Perez-Labrada et al., 2019).

Research demonstrates that nanoparticles provide additive benefits even in tolerant varieties. The combination of inherent tolerance mechanisms and nanoparticle-enhanced defenses produces superior stress protection. Tolerant varieties treated with nanoparticles under salt stress often outperform even non-stressed controls, suggesting that nanoparticles can optimize plant performance beyond baseline tolerance (Perez-Labrada et al., 2019).

The mechanisms underlying these additive effects involve complementary actions. Inherent tolerance mechanisms may focus on ion homeostasis and osmotic adjustment, while nanoparticles particularly enhance antioxidant systems and photosynthetic protection. This complementarity allows both endogenous and exogenous protective systems to contribute to overall stress tolerance (Rahul et al., 2025).

### 8.2 Salt-sensitive varieties

Salt-sensitive varieties like PT3 lack effective endogenous stress tolerance mechanisms, making them more dependent on external interventions. Nanoparticle treatments show particularly dramatic effects in sensitive varieties, providing protective mechanisms these plants cannot generate independently (Perez-Labrada et al., 2019).

The magnitude of improvement in sensitive varieties can be striking, with nanoparticle-treated plants showing growth and physiological parameters approaching those of untreated tolerant varieties. This transformation suggests that nanoparticles can partially compensate for genetic deficiencies in stress tolerance mechanisms. However, treated sensitive varieties typically still underperform compared to treated tolerant varieties, indicating that endogenous mechanisms remain important (Rahul et al., 2025).

Nanoparticle treatments could enable cultivation of otherwise unsuitable varieties in salt-affected areas. This could expand variety choices for farmers, allowing selection based on yield potential, fruit quality or market preferences rather than being constrained to the limited pool of salt-tolerant varieties.

## 9 Environmental safety and ecosystem impacts

Nanoparticle behavior like adsorption to soil particles, interaction with soil organic matter, effects on soil microorganisms require thorough investigation. Soil type, pH, organic matter content and microbial community composition all likely influence nanoparticle fate and effects. Understanding these interactions is essential for predicting environmental impacts and optimizing application strategies (Bakshi and Kumar, 2021).

## 10 Regulatory and standardization issues

The lack of standardized protocols for nanoparticle characterization, application and effect assessment hinders comparison across studies and development of best practices. Regulatory frameworks for agricultural nanoparticle use remain underdeveloped in most jurisdictions, creating uncertainty for potential users and developers. Establishing science-based standards and regulations represents an important need (**Table 2**) (Guru et al., 2025).

**Table 2:** Regulatory status of agricultural nanomaterials worldwide.

Region/country	Regulatory framework status	Key regulatory body/initiative	Current focus and guidelines	Challenges and future needs
USA	Case-by-case under existing laws (TSCA, FIFRA, FFDCA); No specific nano-law	EPA, FDA, USDA	Risk assessment based on chemical substance properties; Significant New Use Rules (SNURs)	Lack of standardized definitions and testing protocols; Uncertainty for developers
European Union	Precautionary principle; Specific nano-provisions in existing regulations (REACH, Novel Foods, Biocides)	EFSA, ECHA, European Commission	Hazard and exposure assessment; Explicit definitions and labeling requirements; Safety testing	Implementation complexities; Defining ‘nano’; Adapting test methods for nanomaterials
China	Developing specific national standards and guidelines; Top-down approach	Ministry of Agriculture and Rural Affairs (MARA), Standardization Administration	Standardization of characterization methods; Safety evaluation of nano-pesticides and fertilizers	Enforcement of standards; Balancing innovation with safety concerns
India	Emerging framework; Draft guidelines and policies under development	Department of Biotechnology (DBT), ICAR, FSSAI	Biosafety guidelines for agri-nanotechnology; Encouraging research while drafting regulation	Need for final, comprehensive policy; Capacity building for risk assessment
Brazil	Developing framework; Relies on existing health and environmental laws	ANVISA, IBAMA, MAPA	Risk analysis for novel foods and inputs; Focus on environmental impact	Infrastructure for characterization and testing; Specific nano-regulation development

TSCA, Toxic Substances Control Act; FIFRA, Federal Insecticide, Fungicide and Rodenticide Act; FFDCA, Federal Food, Drug and Cosmetic Act; REACH, Registration, Evaluation, Authorization and Restriction of Chemicals; EFSA, European Food Safety Authority; ECHA, European Chemicals Agency; ICAR, Indian Council of Agricultural Research; FSSAI, Food Safety and Standards Authority of India; ANVISA, Brazilian Health Regulatory Agency; IBAMA, Brazilian Institute of Environment and Renewable Natural Resources; MAPA, Ministry of Agriculture, Livestock and Food Supply (Brazil).

## 11 Implications for sustainable agriculture

### 11.1 Climate change adaptation

Climate change is increasing the prevalence and severity of salinity stress through sea level rise, altered precipitation patterns and increased reliance on lower-quality irrigation water (Dasgupta et al., 2015).

Nanoparticle-based stress tolerance enhancement could play important roles in adapting agriculture to these changes. The potential of copper nanoparticles to enhance tolerance to multiple stress types-not only salinity but also drought, heat and oxidative stress-suggests broad spectrum protective effects valuable in changing climate (El-Tanbouly et al., 2025; Shaikhaldein et al., 2024). This versatility could reduce vulnerability to unpredictable climate patterns and combined stress scenarios increasingly common under climate change (Van Nguyen et al., 2022).

### 11.2 Nutritional security

Maintaining tomato production in salt-affected regions contributes to food security and nutritional security. Tomatoes provide essential vitamins, minerals and phytochemicals, making production stability important for human nutrition (Egea et al., 2023). Nanoparticle technologies that enable production on marginal lands could expand cultivated area without encroaching on natural ecosystems (Haq et al., 2024).

The potential for enabling cultivation of high-quality, high-yielding varieties in salt-affected areas could improve both production quantity and nutritional quality. This flexibility in variety selection represents an often-overlooked benefit of stress tolerance technologies (Parra-Torrejon et al., 2023).

### 11.3 Sustainable development goals

Nanoparticle applications for stress tolerance align with multiple Sustainable Development Goals (SDGs), including Zero Hunger (SDG 2), Clean Water and Sanitation (SDG 6), Climate Action (SDG 13) and Life on Land (SDG 15). By improving agricultural productivity under challenging conditions, reducing water quality requirements, supporting climate adaptation and potentially reducing agricultural land expansion, nanoparticle technologies could contribute to sustainable development objectives (Jassim et al., 2025; Ur Rahim et al., 2021; Verma et al., 2022; Zafar et al., 2024).

However, realizing these benefits requires responsible development and deployment, with careful attention to potential risks and unintended consequences. Inclusive development processes that consider diverse stakeholder perspectives-farmers, consumers, environmentalists, regulators will be essential for socially and environmentally sustainable implementation.

## 12 Conclusion

It is amply clear that salinity is a major environmental stress limiting tomato (*Solanum lycopersicum* L.) production worldwide. The detrimental effects of salinity are manifested through osmotic imbalances, ion toxicity and oxidative damage; leading to impaired growth, photosynthesis and yield component. Copper-based nanoparticles (Cu-NPs) are emerging as promising nano-agrochemicals, as they can mitigate the adverse effects of stress by modulating several of these cellular processes simultaneously, in contrast to most conventional inputs that target single pathways.

Scientists have reported that Cu-NPs strengthen the antioxidant capacity, help Na^+^ exclusion and vacuolar sequestration, support K^+^ and Ca²^+^ homeostasis and also stabilize chlorophyll content, photosystem II efficiency and gas exchange under saline conditions. Physiological and biochemical measurements indicate that Cu-NP-treated tomato plants maintain more favorable K^+^/Na^+^ ratios, relative water content and carbon assimilation than untreated, salt-stressed plants. Proteomic analyses strengthens the hypothesis that Cu-NPs act through network-level reprogramming of cellular homeostasis through coordinated modulation of proteins involved in redox regulation, ion transport, photosynthesis, primary metabolism and stress signaling. Together, these multi-scale data provide a mechanistic explanation for the improved growth and yield observed under salinity.

However, use of Cu-NPs is strongly concentration dependent. Their efficacy and safety depend on nanoparticle properties (size, oxidation state, surface chemistry etc) and dosage, along with the intensity and duration of salinity stress. Most available literature supports an optimal window in which standardized Cu-NP concentrations under moderate salinity confer maximal benefit, whereas higher doses can cause copper over-accumulation, renewed oxidative stress and growth inhibition.

Genotype-specific responses, with salt-tolerant and salt-sensitive cultivars differing in magnitude and components of Cu-NP mediated tolerance, indicate that nano-enabled interventions should complement, not replace, breeding for salinity tolerance.

Critical knowledge gaps currently limit field-scale deployment of Cu-NPs. Uptake routes, transformations and long-term storage forms of Cu-NPs in tomato and rhizosphere compartments are yet to be resolved under actual soil conditions. The cumulative impacts of repeated applications on soil physicochemical properties, microbial communities, non-target organisms and food-chain copper loading are poorly quantified. In parallel, the absence of harmonized protocols for nanoparticle characterization, exposure metrics and biological endpoints hinders robust cross-study comparison and risk-benefit assessment.

Future work should therefore use an integrated multi-omics (genomics, transcriptomics, proteomics and metabolomics) approach to map Cu-NP-dependent stress-response networks; and couple these approaches with advanced imaging and nano-sensors for spatio-temporal monitoring of plant status. Multi-location field trials across contrasting genotypes and salinity regimes can help define agronomically relevant dose–response relationships. Addressing these priorities is essential to position Cu-NPs as safe, effective and sustainable components of integrated strategies for tomato cultivation on salt-affected lands.
